# Neurobiological Consequences of Sleep Deprivation 

**DOI:** 10.2174/1570159X11311030001

**Published:** 2013-05

**Authors:** Karim Alkadhi, Munder Zagaar, Ibrahim Alhaider, Samina Salim, Abdulaziz Aleisa

**Affiliations:** 1 Department of Pharmacological and Pharmaceutical Sciences, University of Houston, Texas, USA; 2 College of Clinical Pharmacy, King Faisal University, Al-Hofuf, Kingdom of Saudi Arabia; 3 Department of Pharmacology, College of Pharmacy, King Saud University, Riyadh, Saudi Arabia

**Keywords:** Modified Multiple Platform, LTP, LTD, electrophysiology, anxiety, neurogenesis, nicotine, exercise.

## Abstract

Although the physiological function of sleep is not completely understood, it is well documented that it contributes significantly to the process of learning and memory. Ample evidence suggests that adequate sleep is essential for fostering connections among neuronal networks for memory consolidation in the hippocampus. Sleep deprivation studies are extremely valuable in understanding why we sleep and what are the consequences of sleep loss. Experimental sleep deprivation in animals allows us to gain insight into the mechanism of sleep at levels not possible to study in human subjects. Many useful approaches have been utilized to evaluate the effect of sleep loss on cognitive function, each with relative advantages and disadvantages. In this review we discuss sleep and the detrimental effects of sleep deprivation mostly in experimental animals. The negative effects of sleep deprivation on various aspects of brain function including learning and memory, synaptic plasticity and the state of cognition-related signaling molecules are discussed.

## 1. INTRODUCTION

Deficits in cognitive function, as a consequence of sleep loss are particularly prevalent in modern societies where social and occupational demands make it imperative to sacrifice sleep, to increase productivity. These deficits are especially detrimental when sleep loss is chronic, directly causing disruption in the learning and memory processes at the cellular level. This may imply that sleep plays an essential role in cognitive functions such as attention, emotion and memory, independent of any physical manifestations of sleep loss such as sleepiness or drowsiness [1]. 

The idea that the function of sleep is merely to restore exhausted brain metabolism offers a limited view into the complex processes that occur in the sleeping brain [2,3]. Expert consensus indicates that sleep is an active phenomenon that incorporates brain regions involved in information processing during wakefulness. This is suggested by experiments that show an increase in sleep amount after learning [4] and improved memory after rapid eye movement (REM) sleep [5,6], together with the presence of certain waking brain activity patterns during REM sleep [7,8]. Interestingly, current evidence further suggests that sleep may be a privileged time window, free of interference from external sensory inputs, that allows the brain to consolidate newly acquired memories [9,10]. 

The idea that sleep is involved in memory processing dates back to the 19^th^ century with the belief that dreaming could alter associative memories acquired prior to sleep. The general view today is that these two processes are in fact dynamic chemical processes consisting of various types and stages that interact extensively to influence brain functions [11,12]. In fact, it is likely that different stages of sleep are important to certain phases of memory, which are also processed across various brain regions. A particular emphasis has been placed on the hippocampus, where sleep both before and after learning, has been shown to contribute to episodic memory consolidation and neuronal plasticity [10,13-17]. Episodic memory is considered explicit, or declarative memory, which involves conscious and intentional recall of faces, spatial arrangements, objects, and events [18]. This type of memory primarily relies on the hippocampus and associated medial temporal lobe structures as well as neocortical areas for long-term storage [10,18-20]. Several excellent reviews have discussed in great length, some of the highly complex core issues concerning the effects of sleep deprivation (SD) on several aspects including cognitive function, role of stress, sleepiness, role of circadian disruption, and distinctions between human and animal studies [21,22]. Our intention is not to re-visit the same ground already covered extensively by experts who have contributed immensely to sleep literature, instead, our primary purpose is to provide an overview for molecular neuroscientists seeking a concise account of SD effects on learning and memory and associated signal transduction mechanisms. Herein, we discuss sleep deprivation and its detrimental effects on various aspects of brain function including learning and memory, synaptic plasticity and cognition-related signaling enabled *via *key signal transduction molecules. 

## 2. SLEEP, SLEEP DEPRIVATION AND BRAIN FUNCTION

Sleep, like other physiological processes, is regulated by circadian timing systems in the hypothalamus and hallmarked by the cyclic occurrence of two main phases: Non-rapid-eye movement (NREM) sleep followed by REM sleep [23-25]. Non-REM sleep includes lighter sleep stages 1 (N1) and 2 (N2) followed by the deeper, more restful, slow-wave sleep (SWS) or N3 (formally; stages 3 and 4). Polysomnographic studies in humans were the first to definitively show that different sleep stages manifest and alternate regularly in a composed manner classified as sleep cycles. A complete night of sleep consists of five to six major cycles, and although all sleep cycles in a night last about 90 minutes, the duration of each of the two major sleep phases changes as the night progresses with REM sleep increasing and NREM sleep decreasing [26]. The deepest sleep occurs during stage N3 NREM sleep and is characterized by increased parasympathetic tone and an EEG dominated by large amplitude, low frequency delta waves. In contrast, REM sleep is a period of increased brain activity similar to that observed during wakefulness and is hallmarked by events including a generalized loss of skeletal muscle tone that occurs in temporal association with rapid eye movements [26]. The cyclical occurrence of sleep is well structured, as each sleep stage possesses a particular chemical, cellular and anatomical signature [26]. 

Sleep shares similarities between humans and animals but there are also distinct differences that may offer insight into the function of sleep. For instance, in adult mammals, there is a cyclic alternation between sleep and wakeful states similar to humans, where NREM sleep always precedes REM sleep, although the alternation between sleep states is more rapid [27]. Sleep stage amounts, daily distributions and temporal factors of ultradian rhythm remain largely species-specific but tend to be directly correlated to the size of the animal and inversely related with basal metabolic rate [27]. Furthermore, depending on the predation risk, prey animals such as birds do not generally engage in lengthy bouts of REM sleep due to its characteristic muscle atonia and high arousal threshold [28]. Also, sleep structure in reptiles, amphibians and fish is more ambiguous in that even though there is an apparent absence of electrophysiological correlates of sleep, they still show behavioral signs of a homeostatic sleep-like state. Another distinctive feature found in aquatic mammals and birds is unihemispheric SWS, a unique state where one cerebral hemisphere shows waking electroencephalographic (EEG) activity while the other simultaneously shows SWS activity. This feature is thought to reconcile the need for sleep and breathing in aquatic mammals while maintaining vigilance against predators [29].

Regardless of the species however, the physiological need for sleep is undoubtedly one of the strongest drives found in animals and humans, which is best signified by the complex yet redundant neurochemistry involved in sleep. Such a process must be vital if it is worth the risk of decreased vigilance and vulnerability to predatory attack. Accordingly, the detrimental effects of SD on different physiological aspects such as: immunological factors, body temperature regulation, endocrine secretion patterns and learning and memory processes are thought to stem from homeostatic imbalance [31]. It is thought that as the organism repeatedly attempts to restore homeostasis by trying to sleep throughout the period of forced wakefulness, the stress of being continuously awake becomes greater and an “allostatic load” develops [26,30]. The allostatic load represents a change in the set point for homeostasis and if left unchecked can lead to structural and functional remodeling of brain regions involved in memory such as the hippocampus [26,30].

Although the structure of sleep has been well characterized in animals and humans, the physiological function of sleep is still not well understood. One of the most intriguing hypotheses is that sleep makes fundamental contributions to memory processes. Indeed, numerous studies show that sleep could contribute to the consolidation of memory and synaptic changes after initial induction of plasticity during wakefulness [31-34]. In fact, SD studies have been useful in showing that sleep can modulate excitatory synaptic transmission and N-methyl-D-aspartate (NMDA) receptor-dependent signaling in the hippocampus [35-38], which may create the molecular milieu conducive to both functional and structural synaptic plasticity [39-41]. In view of that, a functional imaging study in humans has shown that after a learning task, hippocampal activity increases during sleep [43], while in animals sleep prior to learning can enhance hippocampus dependent memory [44].

Evidence suggests that, certain neuromodulators and cellular processes i.e. gene expression, protein translation mediate the reactivation of previously encoded memory traces and their eventual consolidation in the neocortex. Much of the literature supports a long-term integrative or consolidative role for different phases of sleep in recently acquired memory [15,44]. During NREM sleep, there is synchronized and graded activity between thalamocortical neural networks, which is thought to conduct the initial integration of new memories into a pre-existing long-term memory neural network in the neocortex [10,41]. This process could involve selective reactivation of a particular neuronal network rather than all indiscriminately to prime proper integration of new information on each NREM cycle, specifically at the depolarizing peaks of EEG delta waves. After this initial stage, the subsequent REM sleep phase disconnects the thalamocortical neural networks between the hippocampus and long-term memory stores in the neocortex, which seemingly allows for local NMDA dependent synaptic consolidation processes to take place in these brain regions [10]. This process of selective synaptic strengthening or weakening could serve to better interweave the newly consolidated memories into the existing network and effectively tighten the newly reformed long-term memory network [42]. 

Much of the evidence for the influence of sleep on memory processes is derived from examining the effects of sleep loss in humans and in animals [34,45-47]. In this respect, sleep loss or deprivation is usually defined as experimentally induced lack of sleep for a certain period of time [1]. Total SD seems to be especially detrimental to brain function in humans and animals; whether it is executive decision making [45], categorization [47], spatial memory [17,48] or contextual fear conditioning [49]. This seems particularly relevant in the context of certain occupations in modern societies e.g. medical interns, airline pilots, the military etc. where lack of sleep is sometimes forced at a time when accurate and timely mental performance is crucial to operational effectiveness or even survival [45,50]. Under such circumstances, performance usually depends on attention to the spatial context at hand and the ability to adapt accordingly, which is primarily provided by the hippocampus in conjunction with higher brain areas. 

## 3. ANIMAL MODELS OF SLEEP DEPRIVATION

Many protocols have been developed for experimental SD each with relative advantages and disadvantages. However, there seems to be a common theme across all models to differentiate the stress of forced wakefulness from that of the hypothalamic-pituitary-adrenal (HPA) axis response [51]. With that said, it is important to note that just as there are different stages of sleep, there are also different models that claim to selectively abolish a particular stage of sleep. However, there is some uncertainty as to the validity of these methods in modeling human sleep loss, as it is complicated by other factors such as sleep apnea or insomnia, which is difficult for animal models to mimic. Despite this, sleep apnea and insomniac patients who show fragmented sleep and long sleep latencies, respectively, exhibit stress symptoms such as elevated blood levels of cortisol and epinephrine [52,53].

### 3.1 Forced Activity Model 

One of the oldest forced locomotion SD models utilized to abolish total sleep (REM and NREM cycles) for an extended period is the rotating disk over water model. This involves the implantation of electrodes for EEG monitoring and consists of a metal disk suspended over a shallow pool of water. To control for non-specific environmental confounders, a yoked-control animal that does not come into contact with the water is positioned opposite to the sleep-deprived animal. The electrodes are connected to a computer which detects when the animal enters a prohibited sleep phase upon which the disk is rotated at a low speed of 1.33 rpm and the animal awakens and walks along the disk to avoid falling into the water [54,55]. In this case, the yoked control would also have to walk opposite the disk movement and may also be sleep deprived but from an unspecified sleep phase. Forced locomotion paradigms, such as the treadmill method, are very effective SD methods and have been shown to reduce total sleep to around one tenth of baseline after just 24 hours of total SD [54]. 

The treadmill model that has gained popularity in recent years involves placing animals on a treadmill that moves to awaken the animal upon sleep onset or whenever a prohibited sleep phase is entered. In order to sleep-deprive animals, the forced treadmill paradigm applies forced locomotion 3 seconds on and 12 seconds off while it applies forced locomotion for 15 minutes on and 60 minutes off for control animals. Thus, this ensures both groups walk the same distance but that the controls are able to rest more than their counterparts [56,57]. However, this model may cause fatigue as the animals frequently lose muscle tone after SD, which can confound behavioral observation [58]. Also, the very fact that both the rotating disk and treadmill techniques depend on forced activity can lead to possible confounder stress [54,55,57,59]. Such a bias is evident in the forced treadmill model in that control animals tend to engage in more physical activity compared with their sleep-deprived counterparts, which represents a physical stressor that is fundamentally different than REM SD [51]. 

### 3.2 Gentle Handling Model

Although it is virtually impossible to selectively block one particular phase or stage of sleep without affecting the others, partial SD models offer a useful method to acutely abolish parts of the sleep cycle for relatively shorter periods of time. The gentle handling paradigm involves introducing tactile, olfactory or visual stimulus when the animals enter a particular sleep phase (REM or NREM) monitored by EEG recordings or visual observation [60-65]. This paradigm is useful for shorter deprivation periods e.g. 2-8 hours, because it requires dedicated and continuous supervision by the examiners. There is also the issue of animals adapting to the novel stimuli, which significantly limits the duration of sleep loss [66]. In addition, introduction of novel stimuli to an environment may not only keep animals awake, but it becomes a potential confounder by enriching the animal’s environment and perhaps affecting subsequent behavioral performance [67]. 

### 3.3. Head Lifting Model

A more recent technique, known as the head-lifting method, is believed to be more specific for REM SD. This SD method is not applicable for long durations or multiple animals at once as it requires the researcher to recognize the onset of REM sleep by monitoring polysomnographic recordings of an animal housed in a different room. Within 2-3 seconds of REM onset the researcher pushes a mechanical lever connected to the rat through a series of pulleys and a flexible wire, which awakens the animal by gently lifting its head [68]. This method is useful in abolishing REM sleep episodes within seconds, making it relatively REM sleep specific, although it is a laborious and time consuming method. Additionally, this method is neither applicable for long durations of time nor useful for multiple animals.

### 3.4 Enlarged Environment

The enlarged environment method was developed to accomplish total SD up to about four hours. In this method, sleep loss is carried out by stimulating the animal’s natural tendency to explore novel surroundings by providing the animal with an enlarged environment. This method seems to be stress-free since levels of corticosterone were shown to be unaltered after sleep loss using this method [36]. However, similar to the novel objects exposure method, the possible confounding influence of the enrichment inherent in the enlarged environment method on the learning process of behavioral tasks cannot be overlooked [68]. 

### 3.5. The Modified Multiple Platform Technique

Also known as the “water tank” or “columns-in-water” or “inverted flowerpot” paradigm, the modified multiple platform was designed to achieve mainly REM SD. In this respect, this method is effective in suppressing about 95% of REM sleep, but can interfere with up to 40% of NREM sleep as well [51,69,70]. The prototype of the multiple platform technique was the single inverted flowerpot method, first used by Jouvet and colleagues [71], which consisted of a single narrow platform or upside down flowerpot immersed to about 2 cm above the water level in a water tank [71]. Upon entering REM sleep and losing muscle tone, the animal on the platform subsequently comes in contact with the water and awakens [73-76]. The multiple platforms method was developed to alleviate forced immobility associated with the single platform technique, which allows for greater freedom of movement. This technique was later expanded into the less intrusive modified multiple platform method, allowing multiple animals from already socially stable cages to be sleep-deprived together on multiple columns [75,77]. This is thought to simultaneously address psychosocial, immobilization and isolation stress often observed in the previous versions of the technique [70,75]. In this respect, cage-mates that are sleep-deprived together as socially stable groups have decreased levels of stress hormones such as plasma corticosterone and ACTH compared to socially unstable groups [75]. Also, in an effort to separate the effects of REM SD from other stressors, numerous different functions have been compared. For instance, Na-K-ATPase activity in the brain stem, cerebellum and cerebrum is increased by REM SD, while stressors such as immobility stress and swimming stress do not, which seems to support the distinctiveness of the effects of this type of sleep loss [78]. 

## 4. EFFECTS OF SLEEP DEPRIVATION ON THE CENTRAL NERVOUS SYSTEM

As SD experiments play a key role in elucidating the functions of sleep, many of the first studies documented characteristic behavioral changes after prolonged wakefulness. In the late 1800s, sleep researchers began to keep animals and humans awake and monitored the subsequent effects to infer the function of sleep from these observations [79]. These first studies, mainly performed with dogs, cats and humans, did not possess adequate controls for the methodology used and therefore produced largely inconsistent findings ranging from central nervous system abnormalities to metabolic disruptions including anxiety, body temperature changes and weight gain [80]. Decades later, Dement and colleagues would focus on the psychiatric consequences of REM SD in animals and humans. Results from these studies suggested that REM sleep was strictly homeostatically regulated as evidenced by the more frequent attempts to enter into REM sleep and a robust REM sleep rebound during recovery [81]. Most notably, these studies also observed behavioral disturbances such as difficulty concentrating, anxiety and irritability [82,83]. Both animal and human studies have confirmed that sleep plays an important homeostatic role and that chronic SD is a potent stressor that leads to metabolic and cognitive disturbances in brain areas involved in learning, memory and emotion such as the hippocampus, amygdala and prefrontal cortex [30,84]. Furthermore, it seems that under conditions of SD, there is a deactivation and reorganization of executive control in the prefrontal cortex away from focused thinking [85] towards more hallucinatory mentation of the sort observed in psychiatric patients [86,87]. The advent of the EEG allowed for objective monitoring and characterization of brain activity during sleep and prolonged wakefulness as well as the effect of other stimuli on these states. For instance, sleep deprived individuals display fatigue-related elevation in delta and theta wave activity as measured by EEG, which manifests physically as sleepiness, impaired alertness and performance deficits [88]. However, standing upright position attenuates this effect of SD even after 48 hours of SD, seemingly counteracting the negative effects of SD [88]. These findings seem to offer support to anecdotal evidence, implying that upright posture can increase alertness and inhibit sleepiness in individuals who suffer from short or disrupted sleep [89,90].

Both short- and long-term SD imaging studies in humans have shown reduced alertness and impairment in not only simple tasks such as tests of reaction time or attention, but complex tasks as well that test working memory, logical reasoning and decision making [91-93]. Positron emission tomography (PET) studies have shown that 24-72 hours of SD attenuated global brain glucose metabolism, by 6-8% and as much as 15% in the prefrontal cortex, frontal cortex and thalamus, which are key areas involved in mediating attention and higher order cognitive functions [85,94]. However, functional magnetic resonance imaging (fMRI) showed that even after 35 hours of SD, regional cortical activity depended upon the task being performed. For example, arithmetic calculations and verbal memory testing, which normally activate the temporal lobe, caused decreased and increased activity, respectively, in the prefrontal cortex and parietal lobe of the right hemisphere [94,95]. This finding is further supported by EEG studies showing that the dominance of functional cluster activity in the frontal lobe shifts toward the right hemisphere during total SD in humans [96]. Moreover, after partial SD, participants maintain performance on a continuous attention task while simultaneously displaying a relative increase in EEG activity in the prefrontal cortex [97]. Thus, it is possible that this attenuation of SD induced impairment is accomplished by recruiting collateral brain regions such as the prefrontal cortex, which are able to compensate for inadequate task performance by increasing conscious motivation during the course of an experiment.

## 5. EFFECTS OF SLEEP DEPRIVATION ON LEARNING AND MEMORY

Several lines of evidence from animal and human studies suggest that SD adversely affects cognitive functions such as attention, learning acquisition, working memory and reference memory. For example, sleep loss impairs learning ability and hippocampus dependent memory performance in rodents as measured by various behavioral paradigms including the Morris water maze (MWM) [98], the radial arm water maze (RAWM) [99] and the novel arm recognition task [100].

**Fig. (1):**
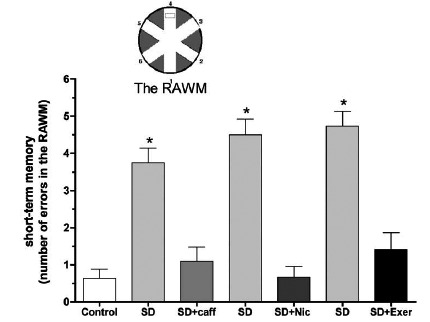
Short-term memory (30 min after training) performance in the radial arm water maze (RAWM) by rats sleep deprived for 24 hour (untreated SD) and/or treated with caffeine (caff), nicotine (Nic) or exercise (Exer). The SD of each group revealed marked impairment of short-term memory indicated by the significantly higher number of errors in RAWM. (*) denotes significant difference (p < 0.001; 10-12 rats/group) from control as well as all treatment groups. Inset: diagram of the RAWM (adapted from: Alhaider *et al*. [108], Zaagar *et al*. [99]).

**Fig. (2):**
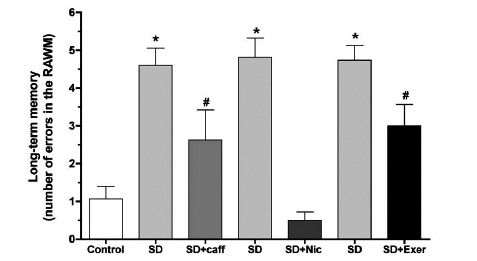
Long-term memory (24h after training and immediately following post-training SD) performance in the radial arm water maze (RAWM) by rats with 24 hour sleep deprivation (untreated SD) and/or treatment with caffeine (caff), nicotine (Nic) or exercise (Exer). The SD of each group revealed marked impairment of long-term memory indicated by the significantly higher number of errors in RAWM. (*) denotes significant difference (p < 0.001; 10-12 rats/group) from all other groups; (#) denotes significant difference from SD as well as control groups (p<0.05; n=10-12 rats). Note that, unlike nicotine treatment, neither caffeine treatment nor exercise is effective in totally reversing the effect of SD. (adapted from: Alhaider *et al*. [108]; Aleisa *et al* [109]).

In particular, SD in rodents has been shown to impair the ability to retain new information and disrupt memory consolidation [34,48,101-105] in proportion to the amount of sleep loss [106]. In this respect, certain increments of time seem to be necessary for consolidation of new memories and their integration onto already existing circuits of the broader memory network. Thus, integration would require coordinated reactivation of the already existing memories affected by the new information through consecutive sleep cycles so as to avoid possible interference from the overlaying of new information [41]. For instance, 6 hours of SD achieved by gentle handling failed to disrupt spontaneous alternation behavior (SAB) in rat whereas 10 hours of SD was enough to decrease SAB in sleep deprived animals compared to controls [106]. This effect can be seen with other behavioral tests such as the MWM, where 6 hours of SD by gentle handling impaired spatial memory, but not learning, and completely failed to influence nonspatial learning or memory in rats [48]. Additionally, even longer bouts, up to 72 hours, of REM SD in rats also showed similar impairment in spatial learning and memory in the MWM [107]. Additionally, in rats that were REM sleep-deprived prior to training in the RAWM, spatial learning acquisition and short-term memory was significantly impaired [99,108,109] (Fig. **1**). It also seems that 24 hr REM SD after RAWM training impairs long term memory (measured at the end of the 24h SD; Fig. **2**). In contrast, other reports have shown that SD had no effects on memory function [110,111]. This discrepancy could be attributed to differences in the type of memory tested, complexity of the task and training, stage of sleep disrupted, duration of sleep loss, number of animals used and the experimental protocols employed. 

These findings are especially pertinent in humans as chronic sleep loss is ever-present in modern societies and primarily hinders brain regions involved in learning and memory processes leading to a significant disruption in brain functioning. 

## 6. SLEEP DEPRIVATION AND ANXIETY

Adequate amount of sleep maintains good mood and cognitive acuity and promotes physiological balance and resilience. Unfortunately, increasing demands of modern societies for vocational or lifestyle reasons leave more people sleep deprived, so much so that SD is no more a one night occurrence but a major public health issue. It is now well known that SD negatively impacts the overall quality of life [112]. SD also is frequently comorbid with several physical and mental illnesses, as either a consequence or a contributing factor, thus increasing the enormity of the impact. Relevant to this, several human studies suggest involvement of SD in development of pathological anxiety [113-118]. Anxiety as a disorder is generally represented by feelings of exaggerated worry, apprehension, uncertainty, fear and tension, affecting an estimated 40 million adults [119,120] in a given year in the United States. Unlike the normal, transient anxiety response caused by a stressful event such as public speaking, pathological anxiety lasts at least 6 months and may worsen if left untreated [120]. In fact, persistent insomnia is reported to be associated with an increased risk of developing anxiety disorder [121]. This highly critical association between sleep disturbance and anxiety begs for an extensive inquiry [122,123]. Consistent with the hypothesis that SD intensifies anxiety, an interesting study by Sagaspe and colleagues [113] reported an increase in self-reported anxiety following a night of SD. Relevant to the present discussion, many animal studies using rodent models of SD also have suggested a causal role of SD in anxiety-like behavior [123-128].

Even though SD and anxiety seem to go hand-in-hand, a clear consensus on whether this relationship is co-relational or causal is clearly lacking. For example, some animal studies have reported an anxiolytic-like effect of SD [74,129-132]. Basically, in these studies REM SD in mice and rats results in increased exploration in the elevated plus maze test and increased exploratory activity in the standard open field test indicating a reduction in anxiety. Some researchers interpret increased locomotion as an anxiolytic-like effect of SD in rodents. However, activity based tasks may not be a true measure of anxiety-like behavior in SD as REM SD is known to cause hyperactivity in some animal models [74] and has even been suggested as a model of mania [133]. 

Several studies have reported that SD decreases anxiety-like behavior while others suggest an increase in anxiety-like behavior. One study reports that, 72 h of REM SD in mice results in anxiogenic behaviors in the elevated plus maze and standard open field tests of anxiety [134]. Furthermore, 72 h treadmill induced SD is reported to increase anxiety-like behavior in open-field and elevated-plus maze tests [127]. SD for 72 h induced by grid suspended over water method caused anxiety-like behavior when tested in mirror chamber, elevated plus maze and zero maze tests [126] in addition to weight loss and impaired locomotor activity [124]. In contrast, other studies report that SD decreases anxiety. REM SD is reported to result in increased exploration in the elevated plus maze indicative of reduced anxiety in mice [135] and rats [132,136]. In addition, REM SD results in increased exploratory activity in open field test [136,137]. 

Later, Pokk and Zharkovsky [129] studied the effect of SD on the behavioral changes caused by diazepam withdrawal in mice. They reported that 12h after the last dose of diazepam, the mice were REM sleep deprived for 24 h on small platform and that this SD paradigm attenuates the anxiogenic effect of diazepam withdrawal when tested in the elevated plus-maze. Pokk and Zharkovskym [130-131] in two other studies reported that SD of mice for 24 h using the small platform technique caused behavioral excitation that was reflected by an increase in the locomotor activity in the elevated plus maze test. Suchecki *et al*. [132], using two models of SD in which rats are REM sleep-deprived either by the classical single platform method or by the modified multiple platform method, report that REM SD induces a differential modification of exploratory behavior in the elevated plus maze. Individually sleep deprived rats exhibit increased locomotor activity, while group sleep-deprived rats display less anxiety-like behavior. Furthermore, stress-induced reduction in open field locomotion is reported to be reversed by 12 h SD during the resting phase, an intervention known to have antidepressant effects in humans [138]. Thus, it is quite apparent that the discrepant interpretations, specifically with regard to the animal models, are most likely a result of variations in the SD protocols used and different anxiety tests employed. Human studies have added to even more confusion. For example, one human study reports an increase in self-reported anxiety after 36 h of SD [113] while another suggests that one night of total SD decreases anxiety in depressed patients with no change of anxiety in patients with panic disorder [118]. Low sample size or lack of data on the complications from previous illnesses or co-occurrence of comorbid diseases seems to be the hidden variable.

It is important to consider that SD is not an isolated problem but more often associated with diseases including hypertension, anxiety disorders or depression as a cause or an effect phenomenon. This makes it hard to isolate symptoms/problems associated solely with SD. While a mechanistic basis for the relationship between SD and anxiety remains largely enigmatic, an emerging link is the involvement of oxidative stress in this process. Vollert *et al*. [139] recently reported that SD is associated with specific changes in oxidative stress and antioxidant enzyme levels as well as alterations in anxiety-like behavior of rats. Interestingly, in this study, treadmill exercise training, through modulation of oxidative stress prevented anxiety-like behavior. In the past, several theories suggest that SD causes oxidative damage, an imbalance between reactive oxygen species (ROS) production and clearance by endogenous antioxidant defense system [134], in discrete areas of the brain [140-142]. This is supported by the fact that sleep is known to decrease oxidative stress [143] by removing oxidants produced during wake time [144]. Therefore, it seems that sleep induces repair, restoration and detoxification [143,144]. 

In humans, regular physical exercise is reported to promote healthy sleep. It is reported to have antidepressant and anxiolytic effects and known to be protective against stress [145-147]. Although the anxiolytic and antidepressant effects of exercise training are relatively mild in normal individuals, greater anxiolytic and antidepressant effects are reported in depressed subjects [148-151]. However, the molecular basis for these beneficial effects of exercise observed in humans remains unclear. Animal studies do offer some insights into these mechanisms. Specifically, the study of Vollert and colleagues [139] shed new light into these largely unknown biological underpinnings of SD associated anxiety-like behaviors in rats and underscore the causal role of oxidative stress in anxiety-like behavior.

## 7. SLEEP DEPRIVATION AND NEUROGENESIS

Hippocampal neurogenesis is a life long process in which newborn neurons continue to mature and integrate in the functional network of the dentate gyrus [152-154]. Adult neurogenesis in certain areas of the brain has been observed in mammalian species examined including humans [155-157]. Any impairment in these processes may lead to pathogenesis of neuropsychiatric and neurodegenerative disorders [158-165].

Sleep is implicated in brain plasticity and mood regulation, hence a functional relationship between neurogenesis, sleep, anxiety, memory and depression seems possible [137,166,167]. Relevant to this, sleep loss is reported to inhibit hippocampal cell proliferation [57] and neurogenesis [168], processes implicated in learning and memory [138,169]. These effects have been produced with multiple days of total SD [170,171], sleep restriction or sleep fragmentation [166, 172,173]. However, shorter durations of SD have produced conflicting results in several rodent studies [174-178].

Some studies suggest that cell proliferation is unchanged by SD [176] while others suggest that acute SD is among the conditions that upregulate hippocampal neurogenesis [177] or that short-term 12 h SD transiently produces more hippocampal progenitor cells *via *cell cycle acceleration [178]. Species differences are likely responsible for the inconsistent observations [179]. A number of investigators have raised the question of whether the effect of SD on hippocampal neurogenesis is an outcome of circadian disorganization rather than loss of sleep per se. This is a valid question since hippocampal neurons show circadian expression of clock genes *in vivo* [180-182], and are exposed to daily rhythms of hormones and neurotransmitters, many of which regulate cell proliferation. Recently, however, a report by Mueller *et al*. [170] suggests that inhibition of hippocampal neurogenesis by SD is independent of circadian disruption, implying involvement of other mechanisms. 

Although consensus on the “theory of neurogenesis” that determines SD-induced regulation of learning and memory is lacking, the exact molecular mechanisms governing this relationship remain an enigma. Several prevailing theories implicate a role of brain derived neurotrophic factor (BDNF) in this process. BDNF is considered a pivotal modulator of neuronal plasticity, neurogenesis, and cognition [183]. Involvement of oxidative stress in SD induced impairment of neurogenesis and consequent learning and memory deficiency is an attractive concept, even though detailed mechanisms of oxidative stress on neurogenesis remain unknown. Moreover, oxidative stress seems to impair neurogenesis [184,185] and antioxidants can reverse this effect [184,186]. Relevant to this, treadmill exercise training or a physical exercise mimetic (e.g. the antioxidant drug tempol) is reported to prevent increased anxiety-like behavior induced by SD [139] or by direct pharmacological induction of oxidative stress [187] in rats. It remains to be seen whether this behavioral protective effect of treadmill exercise or tempol is mediated by increased neurogenesis. Of note is the fact that SD elicits pro-inflammatory effects [188], which compromise overall immunity and good health. The inflammation-induced impairment of neurogenesis *via *action of oxidative stress [189-191] prompts us to speculate an intriguing network of possibilities as illustrated in Fig. (**3**), where oxidative stress appears as a key culprit.

## 8. SLEEP DEPRIVATION PROMOTES INFLAMMATION

It is well known that SD compromises the immune function in humans and experimental animals by provoking a pro-inflammatory response *via *increased cytokine secretion [188,193,194] including IL-1, tumor necrosis factor (TNF) and IL-6 release [195,196]. C-reactive protein (CRP), a molecule produced by the liver in response to inflammation [197,198], increases upon total and partial SD in healthy volunteers [199,200]. CRP is a crucial marker for SD-induced inflammation, as it is not regulated by circadian variation [199]. Thus, all well-known markers of acute inflammatory system including IL-1 beta, TNF-alpha, IL-6 and CRP exhibit responsiveness to sleep manipulation. However, our knowledge of molecular mechanisms that regulate psychological stress-induced inflammation is not very deep. Several mechanisms have been suggested including involvement of vascular mechanisms, altered stress response system, blood pressure and catecholamine upregulation as possible contributing factors to inflammation during SD [201]. Involvement of metabolic systems (insulin resistance) as putative mechanisms for increased inflammation during SD also has been proposed. Additionally, there is evidence indicating that enhanced pro-inflammatory cytokine signaling may promote ROS generation and lead to oxidative damage [189-192]. There is also evidence suggesting that pharmacological induction of oxidative stress leads to increased CRP-1, TNF-alpha and IL-6 [202] and this might be one mechanism that links SD induced inflammation to neuropsychiatric diseases. Therefore, it is imperative to develop a mechanistic understanding of sleep loss-associated inflammation and its impact on mental health.

## 9. SLEEP DEPRIVATION AND SYNAPTIC PLASTICITY 

Of the various forms of synaptic plasticity, long-term potentiation (LTP) and long-term depression (LTD) are the most important. Based on the proposition that memory is encoded by changing the strength of synaptic connections among networks of neurons, both LTP and LTD may have an important role in learning and memory and therefore believed to be the cellular correlates of these functions. 

### 9.1. Effect on LTP

Although the effects of sleep and SD on learning and memory have been studied rather extensively, the impact of SD on hippocampal LTP has just recently been investigated. Hippocampal LTP, a neural substrate of learning and memory [203], has two major types; early phase (E-LTP) and late phase (L-LTP), which correlate with short and long-term memory respectively. Both types have been identified in the CA1 and DG areas of hippocampus and require activation of different protein kinases. Additionally, L-LTP requires synthesis of new protein. The inhibitory effects of SD on hippocampal LTP were predicted based on the finding that SD impaired hippocampus-dependent learning and memory [101,204]. The effect of 12 hours of forced locomotion model of SD on LTP was first reported by Campbell and colleagues [55] who showed impaired LTP in area CA1 of rat hippocampal slices. In another investigation, single platform 24, 48 or 72 hours SD impaired both induction and maintenance of LTP in area CA1 of rat hippocampal slices with greater impairment after 72 hour SD compared to that of 24 hours [205]. Both studies reported elevation in the levels of glucocorticoids in sleep-deprived animals due to the use of a stressful method of SD [55,205]. However, elimination of the adrenal stress response to SD by the corticosterone inhibitor, metyrapone [206] or by adrenalectomy [207] did not prevent the effect of SD on learning and memory. Moreover, when a single large platform is used, which introduces an immobilization stress effect but allows the animal to sleep, it does not show impairment of LTP suggesting that the impairment of LTP during SD is not due to elevated stress hormones [34]. 

**Fig. (4):**
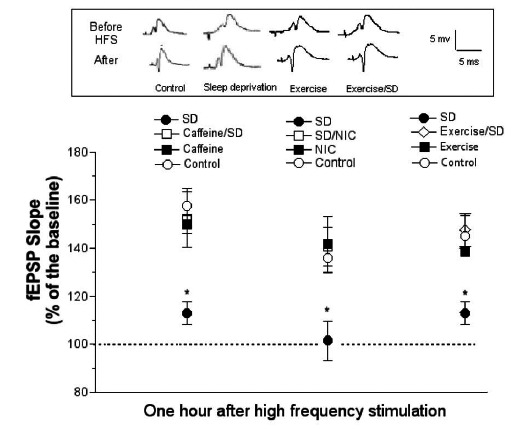
Sleep deprivation (SD) severely suppressed Hippocampal early LTP (E-LTP) represented by increases in fEPSP slope of population spike evoked by HFS of the Schaffer collaterals/commissural synapses in area CA1 of anesthetized rats (measured at 60 min after HFS: 8 pulses at 400 Hz/10s repeated 8 times). Chronic caffeine, nicotine or regular exercise treatment of sleep-deprived rats prevented the deleterious effect of SD. (*) indicates significant difference from all groups (p <0.05-0.001, n=5-7 rats). Inset is population spikes from a representative exercise set of experiments; calibrations, 5 mV/5 ms, apply to all traces. (adapted from: Alhaider *et al*. [108]; Aleisa *et al*. [109]; Zagaar *et al*. [99]).

Sleep deprivation for 72 hours in the modified multiple platform, which eliminates immobilization and isolation stresses, inhibits LTP in both CA1 and DG areas in hippocampal slices and reduces membrane excitability of CA1 pyramidal neurons but not that of the DG granule cells [34]. The finding that SD inhibits LTP of the DG area without impairing membrane excitability of DG granule cells, suggests that the reduction in membrane excitability due to SD is not the main mechanism for the impairment of LTP [34]. In agreement with these studies, recent report showed impairment of LTP in both CA1 and DG areas of urethane anesthetized rats after 24 and 48 h of SD in the modified multiple platform [108]. Furthermore, there was no change in the basal synaptic transmission during SD in CA1 or DG areas [108,208,209]. In these *in vivo* recordings, 24 or 48 h SD caused near total inhibition of E-LTP in both CA1 and DG areas [99,109] (Fig. **4**). Additionally, SD for 24 h caused total inhibition of the L-LTP in both CA1 and DG areas of the hippocampus [209,210].

### 9.2 Effect on LTD

The effect of SD on LTD is variable and needs to be closely investigated. While one study reported a normal LTD but impaired LTP in area CA1 of rat hippocampal slices after 72 hour SD in multiple platform [34], another study in the mouse hippocampal slices reported impaired LTP with an increase in the magnitude of LTD evoked at 5 Hz, but decreased at 1 Hz [36]. More recent reports showed an enhancement of LTD in anesthetized rats [211] or in rat hippocampal slices [212,213]. These contradictory results can be explained by the use of different SD models, different protocol to induce LTD and different species of animals among others. For example; in addition to different animal species and electrophysiological methods used, the SD paradigms used are variable; some investigators used single platform [34,36] while others used gentle handling [211-213] for varying durations of SD. Therefore, whereas SD suppresses LTP in hippocampal CA1 [34-36,109,210,211,214] and dentate gyrus (DG) areas [108,215,216], LTD, induced by paired-pulse facilitation, is not affected after 24 hours of sleep loss [214].

The negative impact that SD has on synaptic plasticity is thought to be a product of the underlying deleterious changes in plasticity-related intracellular signaling. For instance, hippocampal glutamate NMDA receptors, which are critical for LTP induction, show negative alterations in receptor subunit composition and turnover after 24 hours of REM SD. It has been shown that SD reduces the expression of NMDA receptors, decreases the levels of the GluN1 subunit of NMDA receptor and lowers excitatory postsynaptic currents in area CA1 [37]. However, recently, it has been reported that although 72 hours of SD decreases the expression of GluN2B receptor subunit in the frontal cortex and hippocampus, it does not change GluN1 subunit [217]. Thus, it seems that the duration of SD may have differential effects on NMDA receptors. Interestingly, after 30 days of 20 hours/day sleep restriction, there was less NMDA-induced excitotoxicity in these rats [218]. This is probably due to loss of neurons as well as decreased number of NMDA receptors. In addition, 12 hours of SD impairs both phosphorylation and membrane trafficking of hippocampal glutamate alpha-amino-3-hydroxy-5-methyl-4-isoxazolepropionic acid (AMPA) receptors, which are crucial in maintaining synaptic plasticity [219]. These changes may affect the activity of several downstream signaling molecules. In particular, the expression of phosphorylated calcium calmodulin kinase (P-CaMKII) is markedly decreased after 24 hours of SD whereas the levels and activity of calcineurin are increased [107,108]. 

## 10. SLEEP DEPRIVATION DISRUPTS COGNITION-RELATED SIGNALING MOLECULES

The negative impact of SD on LTP and synaptic plasticity is thought to be a product of disruptive changes in intracellular signaling molecules and receptors including NMDA [220,221] and AMPA receptors [100]. For instance, molecular studies have shown that the expression of key signaling proteins and trophic factors, e.g. CREB and BDNF, involved in LTP and memory are impaired in the hippocampus after 8, 24, and 48 hours of SD [46, 108]. Also, SD can negatively impact other intracellular signaling pathways such as the cAMP/PKA pathway, which plays an important modulatory role in LTP and memory. Sleep loss, for as little as 5 hours, increases the levels of phosphodiesterase IV, which in turn impairs LTP expression and decreases the levels of cAMP [222]. We have shown impaired synaptic plasticity in various brain disorders to be correlated with impaired levels of P-CaMKII and P-CREB during expression of E-LTP and L-LTP, respectively [223-226]. In the following sections we briefly discuss the effect of SD on a number of signaling proteins essential for expression of synaptic plasticity. 

### 10.1. Calcium Calmodulin-dependent Protein Kinase II (CaMKII)

CaMKII is critical in the expression of LTP in the hippocampus [227-230]. The negative impact of SD on memory is thought to be mediated by deleterious changes in intracellular signaling indicated by the down-regulation of basal and stimulated P-CaMKII protein expression in both the CA1 and DG areas [108]. Other molecular studies support this conclusion showing that the expression and activation of CaMKII are impaired in the hippocampus after various durations of SD [46,208]. For instance, the expression of P-CaMKII during E-LTP is markedly decreased after 24 hours of SD whereas the level of the phosphatase calcineurin, which dephosphorylates CaMKII, is increased [108,208]. Sleep deprivation for 24 hours reduced the ratio of P-CaMKII:total CaMKII at the basal and stimulated levels in the DG area, which may indicate that SD mainly hinders the phosphorylation process of CaMKII under these conditions [99,108]. In area CA1, however, the ratio of basal P-CaMKII:total CaMKII was largely unchanged across the groups, implying that SD primarily reduces P-CaMKII levels by decreasing the overall synthesis of CaMKII. The down regulation of P-CaMKII by SD (Table **1**) has far-reaching consequences on other molecules and receptors e.g. AMPA and NMDA receptors that ultimately lead to suppression of synaptic plasticity in the hippocampus [219,220].

### 10.2. Calcineurin (PP2B)

Protein phosphatases are responsible for the dephosphorylation and deactivation processes of previously phosphorylated molecules. Activation of calcineurin (PP2B) by Ca^2+^-calmodulin leads to the activation of protein phosphatase 1 (PP1), which in turn inactivates P-CaMKII [231,232]. Evidence indicates that calcineurin reduces post-synaptic activity and impairs LTP in the hippocampus [233,234]. It has been reported that over-expression of calcineurin in the hippocampus impairs LTP [234] and that pharmacological inhibitors of calcineurin facilitate LTP in hippocampal slices [233].

We have shown that basal protein levels of calcineurin in the total homogenate of the CA1 and DG areas were not affected by 24 h of SD [99,108] (Table **1**). Sleep loss, even for 72 h, does not affect the basal levels of calcineurin in the hippocampus [17]. Furthermore, it has been reported that SD does not influence the level of PP1, which is activated by calcineurin [48]. Although SD does not seem to interfere with the basal protein levels of calcineurin in the CA1 and DG areas of the hippocampus, the possibility that SD may affect only calcineurin activity or the level/activity of another phosphatase cannot be excluded. In fact, the activity of calcineurin has been found to increase after 72 h of SD [107]. Thus, SD seems to enhance the activity of existing calcineurin without affecting its expression. 

### 10.3 Brain-Derived Neurotrophic Factor (BDNF)

Endogenous BDNF plays an essential role in spatial learning and memory as well as hippocampal LTP. Studies in BDNF-knockout mice show impairment in spatial memory [235] and hippocampal LTP [236,237]. Furthermore, the gene expression of BDNF has been reported to be increased in the hippocampus after spatial learning tasks [238,239], and after the induction of LTP [240-244].

BDNF influences synaptic plasticity by raising the efficiency of synaptic transmission through activation of CaMKII and CREB [245,246]. Particularly in the hippocampus, BDNF enhances activation of CaMKII and CREB, probably by a mechanism that involves the release of Ca^2+^ from the internal stores through the tropomyosin kinase B (TrkB) receptor/phospholipase Cγ pathway [245,247,248]. Therefore, it is possible that SD impairs E-LTP by suppressing the increase of BDNF and P-CaMKII at the basal and stimulated levels. Twenty four hour SD reduces the basal levels of BDNF in the CA1 (Table **1**) and DG areas, which is consistent with the finding that 8 and 48 h periods of SD decrease the gene expression and the protein levels of BDNF in the hippocampus [46]. In our studies, the decreased basal levels of up-stream effectors such as BDNF in the CA1 and DG areas of sleep-deprived rats may be considered a contributing factor to the reduction of both P-CaMKII and P-CREB basal levels. Additionally, the SD-induced suppression of BDNF up-regulation after stimulation is indicative of decreased availability of extracellular BDNF [99,108,208,209].

### 10.4 cAMP Response Element Binding Protein (CREB)

CREB is a transcription factor involved in the regulation of CRE-containing genes. Accumulating evidence has shown that expression of L-LTP and long-term memory requires CREB activation [249-252]. Induction of repressor or activator forms of CREB affects long-term memory negatively or positively, respectively, without affecting short-term memory [249,253].

CREB mediates the synthesis of proteins that are important for long-term synaptic plasticity and memory. The gene expression of CREB has been found to decrease after 8 and 48h periods of sleep loss in the hippocampus [46]. This reduction could be attributed to the significant decrease in the upstream modulators of CREB including extracellular signal-regulated kinase (ERK) and calcium/calmodulin-dependent protein kinase IV (CaMKIV). In fact, it has been reported that sleep loss decreases the levels of ERK phosphorylation [48]. Moreover, evidence shows that 24 h SD decreases the basal protein levels of total-CREB and P-CREB in CA1 and DG areas [209] (Table **1**). Furthermore, repetitive stimulation in anesthetized sleep-deprived anesthetized rats fails to induce the expected up-regulation of phosphorylated CREB, which may be responsible for the impairment of L-LTP in the CA1 area of sleep-deprived rats [209]. 

### 10.5. Calcium/Calmodulin-dependent Protein Kinase IV (CaMKIV)

The transcriptional activator CaMKIV is present in the nuclei and cytosol of neurons of various brain areas including the hippocampus [254,255]. Upon repetitive stimulation, the levels of CaMKIV increase resulting in phosphorylation and activation of CREB [256,257]. Mice with mutation in CaMKIV show impairment in L-LTP, long-term memory and CREB phosphorylation in the hippocampus [258,259].

Our published and unpublished findings revealed reduced basal protein levels of CaMKIV in CA1 and DG areas (Table **1**) of the hippocampus of the sleep-deprived rats. However, repetitive stimulation produced the expected up-regulation of CaMKIV in sleep-deprived rats [209]. It seems that reduced basal CaMKIV levels may be a factor that contributes to the reduced levels of CREB phosphorylation in sleep-deprived rats leading to impairment of L-LTP. 

## 11. COUNTERING THE EFFECTS OF SLEEP DEPRIVATION ON LEARNING AND MEMORY

To offset the effects of sleep loss and to sustain performance during periods of sleepiness or prolonged wakefulness, pharmacological measures are often required. Commonly used stimulants, including caffeine and nicotine, have different mechanisms of action and varying side effect profiles. In this section, we review some successful measures to counteract the neurobiological deleterious effects of SD in rodent models. 

### 11.1 Nicotine

Nicotine treatment is known to attenuate the impairment of learning and memory and LTP associated with several mental disorders including Alzheimer’s disease and chronic psychosocial stress [224,260]. Treatment of animals acutely with nicotine (1 mg/kg; subcutaneously, twice/daytime) during 24 or 48 h SD in the modified multiple platform significantly prevented or attenuated SD-induced impairment of learning, short-term and long-term memory as measured in the RAWM (Figs. **1** and **2**). Moreover, extracellular recording of field potentials from CA1 and DG areas of the hippocampus in urethane-anesthetized rats showed that concurrent, acute treatment with nicotine prevented SD-induced impairment of LTP [109,210] (Fig. **4**).

The action of nicotine could be due to a number of possible mechanisms. It has been suggested that nicotine increases the probability of coincidence between presynaptic transmitter release and postsynaptic depolarization, thus raising the probability of LTP induction [261,262]. Furthermore, desensitization of α7-nicotininc acetylcholine receptors (α7 nAChR) reduces the release of gamma aminobutyric acid (GABA) from interneurons, thus indirectly increasing pyramidal cell excitability [263] this, in turn, facilitates LTP by decreasing its threshold of induction [264]. Another possible mechanism by which nicotine may counter the deleterious effects of SD is through BDNF. Nicotine treatment increases BDNF mRNA and protein levels [224,265]. In contrast, SD reduces the expression and the protein levels of BDNF in the hippocampus including the DG area [46,108] and reduces the protein levels of the phosphorylated form of the extracellular signal-regulated kinase 2 (phospho-ERK2), which is a major target of BDNF receptor activation [48]. Therefore, it is possible that nicotine treatment may have prevented the effects of SD on memory and synaptic plasticity by restoring normal levels of BDNF.

### 11.2 Caffeine

Caffeine is a common ingredient in numerous beverages that are consumed on a daily basis worldwide. The consumption of caffeine-containing beverages is known to increase during sleep loss to improve alertness and enhance concentration [266]. Indeed, low doses of caffeine have positive effects on learning and memory [267] and chronic caffeine intake has been shown to alleviate cognitive impairment in different animal models of brain disorders including Alzheimer’s disease [268], Parkinson disease [269], attention deficit hyperactivity disorder [270], age-related cognitive decline [271] and scopolamine-induced amnesia [272].

In sleep deprived rats chronic caffeine treatment (0.3 g/L of drinking water) prevented learning and memory impairment induced by 24 h SD in the modified multiple platform method [108,208] (Figs. **1** and **2**). Chronic caffeine treatment also prevented the SD-induced impairment of E-LTP and L-LTP of the CA1 and DG areas of the hippocampus [108] (Fig. **4**). At the molecular level, chronic caffeine prevented SD-induced deficits in the basal and induced levels of a variety of signaling molecules including CaMKII and BDNF [108,208,209] (Table **1**:see section 10). 

It has been suggested that caffeine may act mainly by inhibiting adenosine receptors [273]. There are at least four adenosine G-protein coupled receptors subtypes: A1, A2a, A2b and A3, of which the A1 subtype is highly expressed in the hippocampus. Adenosine activation of A1 receptors inhibits release of glutamate and suppresses LTP in rat hippocampal slices and thus disrupting the process of learning and memory at the synaptic level [274]. In addition, adenosine stabilizes magnesium ion on postsynaptic NMDA receptors, thus preventing NMDA receptor-mediated excitation [275,276]. It has been reported that SD, through induced production of nitric oxide, enhances extracellular adenosine in the basal forebrain leading to enhanced sleep intensity [277]. Therefore, by antagonizing the effects of adenosine, chronic caffeine may sustain the homeostasis in the brain. 

### 11.3. Physical Exercise

Regular aerobic exercise is a promising non-pharmacological approach that can ameliorate memory impairment in a variety of conditions including brain injury [278-280], and can enhance cognitive function [281], prevent memory decline in aging [282,283], decrease anxiety related behaviors [139,187] and attenuate oxidative stress [284,285,287]. 

In sleep-deprived rats the impact of 4 weeks of regular treadmill exercise on SD induced impairment of spatial learning and memory, synaptic plasticity and related signaling molecules in area CA1 of the rat hippocampus was examined [99]. Rats were regularly exercised on a treadmill and subsequently sleep-deprived for 24 h in the modified multiple platform method. Testing of learning and memory performance in the RAWM showed that although sedentary sleep deprived rats were severely impaired, the performance of exercised sleep deprived rats was normal (Figs. **1** and **2**). Additionally, the markedly impaired E-LTP in the sedentary sleep deprived animals was normal in the exercised sleep deprived group (Fig. **4**). At the molecular level, immunoblot analysis of CA1 area before (basal levels; Table **1**) and after expression of E-LTP indicated that the significant down-regulation of BDNF and phosphorylated CaMKII levels seen in sleep-deprived animals, was prevented with regular treadmill exercise. 

Although the precise mechanism by which regular exercise prevents the deleterious effects of SD on memory and LTP is not clearly understood, neurotrophic factors, particularly BDNF, possess most of the properties that could mediate the beneficial effects of exercise on neuronal transmission and connectivity as well as activity-dependent plasticity. Considerable evidence has shown that by up-regulating BDNF, exercise seems to foster the signaling cascades responsible for synaptic plasticity and cognition [286]. Exercise not only induces the expression of a wide array of plasticity-related genes, but can also up-regulate BDNF by engaging aspects of epigenetic regulation to facilitate BDNF gene transcription [287]. These mechanisms may prepare the synapses structurally and functionally to facilitate activity dependent plasticity in the presence of the SD-induced insult. The protective mechanism of regular exercise against SD-induced E-LTP and L-LTP impairment in the hippocampus is accompanied by increasing levels of BDNF [99] (Table **1**), which through its modulatory effects can enhance activity of various signaling cascades to ultimately prevent the decrease in the levels of P-CaMKII and P-CREB in sleep-deprived rats. 

## 12 CONCLUDING REMARKS

Overall, the work presented in this review generally highlights aspects of SD that have been insufficiently studied namely the effect of SD on the brain at the electrophysiological and neuromolecular levels. Electrophysiological and molecular studies have been carried out in animals and although the immediate application of animal data to humans is clearly inappropriate, evidence obtained from human studies recapitulates the basic themes emerging from animal research. 

The interpretation of SD-induced alterations in brain function is complicated by the variety of SD paradigms and protocols used to induce sleep loss. The complications result from the variable length of time of SD, time of day, and most importantly whether the effect of sleep loss is tainted by stresses resulting from unfamiliar environment, social isolation or movement restriction. Use of different strains in different studies is another complicating factor. However, recent experiments increasingly use SD paradigms and protocols that are designed to limit exposure to these stresses and other complicating factors. Thus, it is important for researchers to study the effect of SD uncontaminated by activation of the hypothalamic-pituitary-adrenocortical axis. This can be successfully done through elimination of the adrenal stress response to SD by treatment with the corticosterone inhibitor, metyrapone or by adrenalectomy [206]. In a different approach, we have shown that stress influences the hippocampal area CA1 differently than the DG area. For example, psychosocial stress impairs LTP in area CA1 without affecting the DG area [288,289]. We measured the effect of SD in both areas using electrophysiological and biochemical methods and found that SD negatively impacts LTP and related signaling molecules markedly, in both the CA1 and DG areas [108,208]. This led us to conclude that the memory impairment, LTP suppression and disruption of related molecular cascades seen in rats sleep-deprived in the columns-in-water method are actually the results of extended wakefulness as opposed to any non-specific stresses as a consequence of being in the aquarium environment.

## Figures and Tables

**Fig. (3):**
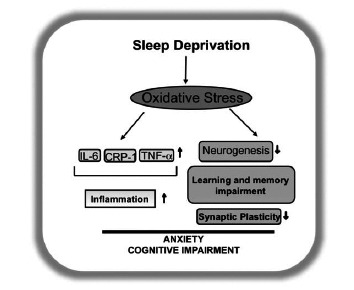
Working hypothesis for the involvement of oxidative stress in the effects of sleep deprivation.

**Table 1.** Summary of the Effects of SD and/or Regular Exercise, or Caffeine on the Basal Levels of Signaling Molecules Important for E-LTP and L-LTP Expression

	SD	Exercise	Exercise/SD	Caffeine	Caffeine/SD
**P-CaMKII**	Decrease	No change	No change	No change	No change
**Total CaMKII**	Decrease	No change	No change	Increase	No change
**Calcineurin**	No change	No change	No change	No change	No change
**BDNF**	Decrease	Increase	Increase	No change	No change
**P-CREB**	Decrease	Increase	Increase	No change	No change
**Total-CREB**	Decrease	No change	No change	No change	No change
**CaMKIV**	Decrease	No change	No change	No change	No change

Protein levels of P-CaMKII, total-CaMKII, calcineurin, BDNF, P-CREB, total-CREB, and CaMKIV in area CA1 of sleep-deprived, exercised, and exercised/ sleep-deprived rats were determined by immunoblot analysis and compared to those of the control rats. All increases or decreases are significantly different from control (p<0.05-0.001; n=5-7 animals) (adapted from: Alhaider *et al*. [99,108,208]).

## ABBREVIATIONS

AMPA: = α-amino-3-hydroxy-5-methyl-4-isoxazole-propionic acid
BDNF: = brain derive neurotrophic factor
CA1: = Cornu Amonis 1
CaMKII: = Calcium calmodulin-dependent protein kinase II
CRP: = C-reactive protein
CREB: = cAMP response element binding protein
DG: = Dentate Gyrus
EEG: = Electroencephalogram
E-LTP: = early phase long-term potentiation
ERK2: = extracellular signal-regulated kinase
L-LTP: = late phase long-term potentiation
LTD: = long-term depression
MWM: = Morris Water Maze
GluN1: = a subunit of NMDA glutamate receptor
GluN2B: = a subunit of NMDA glutamate receptor
NMDA: = N-methyl-D-aspartate
NREM: = non Rapid Eye Movement
RAWM: = Radial Arm Water Maze
REM: = Rapid Eye Movement
ROS: = reactive oxygen species
SAB: = Spontaneous alternation behavior
SD: = sleep deprivation
SWS: = slow wave sleep
TNF: = Tumor necrosis factor
TrkB: = tropomyosine kinase B
